# Perspectives of Nursing Students on Hybrid Simulation-Based Learning Clinical Experience: A Text-Mining Analysis

**DOI:** 10.3390/nursrep14020074

**Published:** 2024-04-18

**Authors:** Aya Saitoh, Tomoe Yokono, Momoe Sakagami, Michi Kashiwa, Hansani Madushika Abeywickrama, Mieko Uchiyama

**Affiliations:** Graduate School of Health Sciences, Faculty of Medicine, Niigata University, Niigata 951-8518, Japan

**Keywords:** COVID-19, nursing education, Japan, nursing students, simulation-based learning

## Abstract

Given the past limitations on clinical practice training during the COVID-19 pandemic, a hybrid format program was developed, combining a time-lapse unfolding case study and high-fidelity simulation. This study assesses the effectiveness of a new form of clinical training from the perspective of student nurses. A questionnaire was administered to 159 second-year nursing students enrolled in the “Basic Nursing Practice II” course. Text mining was performed using quantitative text analysis for the following items: (1) aspects that were learned more deeply, (2) benefits, and (3) difficulties encountered with the new practice format. The new clinical practice format enhanced participants’ learning related to the daily changes required in nursing care and improved their nursing competency through simulated patient interactions. However, the participants faced difficulties dealing with patients accompanied by secular changes. Moreover, they found remote group work challenging. These findings can be applied to the development of new educational strategies.

## 1. Introduction

The COVID-19 pandemic necessitated widespread changes in nursing education worldwide, including in Japan, where universities were compelled to adapt nursing practicums to maintain educational quality and minimize disruptions for students [1,2,3]. In response to the pandemic’s challenges and the need for infection prevention measures, innovative programs integrating information technology, simulations, and online learning emerged. Simulation-based learning emerged as a valuable alternative to traditional clinical practice, offering students opportunities to develop clinical skills, reasoning abilities, and patient care competencies in a safe environment [4]. Notably, Hargreaves et al. [5] demonstrated the adoption of virtual nursing clinical experiences, such as telenursing, coupled with interactive teaching methods like the Teach-Back process, facilitating student engagement and learning. According to a systematic literature review of papers published from 1960 to 2019, hybrid simulation has been shown to provide an excellent training context by enhancing interaction between learners and caregivers, immersing trainees more deeply in the emotions and sensations of scenarios, while also demonstrating effectiveness equivalent to high-fidelity simulators in specific training scenarios [6].

During the fiscal years 2020 and 2021, Niigata University in Japan faced difficulties securing clinical training placements for Basic Nursing Practicum II due to restricted access to hospitals during the pandemic. Consequently, novel approaches were explored to achieve the intended learning outcomes in isolated settings. Consequently, a hybrid high-fidelity simulation utilizing a time-lapse unfolding case study format was developed, combining both on-campus face-to-face and online non-face-to-face components.

The necessity of further research to assess the efficacy of new teaching modalities introduced during the pandemic cannot be overstated. Ensuring that nurses maintain clinical competence and confidence in their skills amidst changing educational landscapes is paramount. Moreover, the evaluation of novel learning approaches is essential for establishing robust practical training programs resilient to pandemics, natural disasters, and similar challenges. Thus, this study aims to evaluate the effectiveness of a hybrid high-fidelity simulation clinical training program from the learners’ perspectives.

## 2. Materials and Methods

We adopted a quantitative descriptive study using a convenience sample of 159 second-year students registered for the “Basic Nursing Practicum II” from 25 February to 6 March 2020 and from 22 February to 5 March 2021 at the Department of Nursing at Niigata University. When selecting our research subjects, we excluded students with clinical experience and focused solely on undergraduate students. Although we did not specifically inquire about the age of participants, it is worth noting that approximately 99% of undergraduate participants were recent high school graduates, with 19-year-old students comprising the majority. Regarding sex, we made a deliberate decision not to conduct separate analysis based on gender, considering the relatively small sample sizes of male students in both the 2020 and 2021 cohorts (4 and 3, respectively), and the lack of significant differences in the gender ratio between the years. A survey was administered electronically using the web-based questionnaire function of the Academic Affairs Information System. Voluntary responses were requested from all students on the last day of the practical training. Students were informed about the purpose and methods of this survey study and that their participation and answers would not affect their grades.

The questionnaire comprised seven items agreed upon by the research team, three of which were single choice, pertaining to whether they (1) understood the changing conditions of patients over time, (2) thought the training would be useful in actual clinical situations, and (3) were motivated to continue their studies and pursue nursing in the future.

The remaining questions were open-ended and asked (4) what the participants learned more about or gleaned a deeper understanding of through the training, (5) positives of the training, and (6) difficulties encountered during the training period (Supplementary Materials S1). The questionnaire was developed by extracting items from previous research and then refining them with input from multiple instructors involved in the practical training.

### 2.1. Outline of the Practical Training (Supplementary Materials S2)

Basic Nursing Practicum II aims to apply learned knowledge, skills, and attitudes in a clinical setting and assist patients with their basic needs. The objectives are to: (1) understand nursing, (2) analyze patient case studies to grasp the nursing process, (3) apply assessment and daily living assistance techniques based on patient conditions, and (4) practice nursing care attitudes and simulations. During the pandemic, training was switched to a hybrid program combining on-campus and online components. A case study of a patient with “community-acquired pneumonia” simulated a realistic situation. The simulation was conducted as an unfolding case study to facilitate a more realistic learning experience. A mock patient simulated the daily changes in her condition as set up in the case study. Simulated electronic medical records, including a “flow sheet (progress record)”, “nursing record”, and “laboratory test results”, were delivered daily. To develop students’ ability to “think like a nurse” in realistic situations, students were encouraged to utilize Tanner’s clinical judgment model to “notice”, “interpret”, “respond”, and “reflect” [7,8]. The new clinical training program was designed to support critical thinking by allowing the students to grasp, judge, and practice situations in real time.

In the first week, students gathered information by visiting the mock patient and conducting the assessment, consequently identifying nursing problems. In the second week, they clarified the nursing problems and formulated a nursing plan by considering the individuality of the mock patient case and using subject, object, assessment, and plan criteria. On the last day, the students delivered a group presentation on “Nursing care based on the individuality of patients”, summarizing what they had learned and experienced during the two-week training period. Hybrid simulation-based learning combines the benefits of traditional clinical experiences with simulation technology. By integrating simulation-based learning into clinical education, nursing students can develop essential clinical skills in a safe, standardized, and accessible environment, ultimately enhancing their readiness for real-world patient care.

### 2.2. Analysis Methods

Descriptive statistics were used for single-choice items, whereas the responses for open-ended questions were analyzed using the text mining method. Higuchi’s [9] KH Coder version 3 for quantitative text analysis was used as the text mining tool. First, the free text responses were converted into text data, and the smallest units of words were extracted. Second, terms that were not segmented appropriately were compared with the raw data and set as forced extraction terms to reflect the intent of the source text. Third, a co-occurrence network analysis was conducted to identify terms with similar appearance patterns or co-occurrence relationships, and subjects were extracted and categorized regarding their co-occurrence. Words with strong co-occurrence relationships were connected by lines, whereas more frequently appearing words were indicated by larger circles. After selecting the “main co-occurring words” in the extracted categories, subgraph names were generated using the KH Coder’s KWIC concordance function to determine how the extracted words were used in the context of free descriptions.

This study was approved by the Medical Research and Ethics Committee of the School of Health Sciences, Niigata University (approval number: 2021-0010).

## 3. Results

Of the 159 respondents (56 out of 79 students in FY 2020; 66 out of 80 students in 2021), 122 (76.7%) completed the questionnaire; the rest were unresponsive. The grade distribution showed that of the 122 respondents, 7 (5.7%) were in the 60–69-point range, 31 (25.4%) in the 70–79-point range, 67 (54.9%) in the 80–89-point range, and 17 (13.9%) in the 90–100-point range.

Most respondents (97.6%) answered that they understood the changes in the patient’s condition over time “very well” or “well”. Most students (80.5%) thought that the training would be useful in actual clinical settings and 115 (94.3%) reported that the training motivated them to continue their studies and learn more (Table 1).

### 3.1. Students’ Learning or Increased Understanding through the Training

In total, 3265 words were extracted from the students’ responses regarding their increased understanding throughout the training period, from which 150 frequently occurring words were selected. The five most frequent words mentioned in responses were: “patient” (209), “nursing” (97), “care” (74), “individualized” (73), and “change” (53) (Table 2).

**Figure 1:**
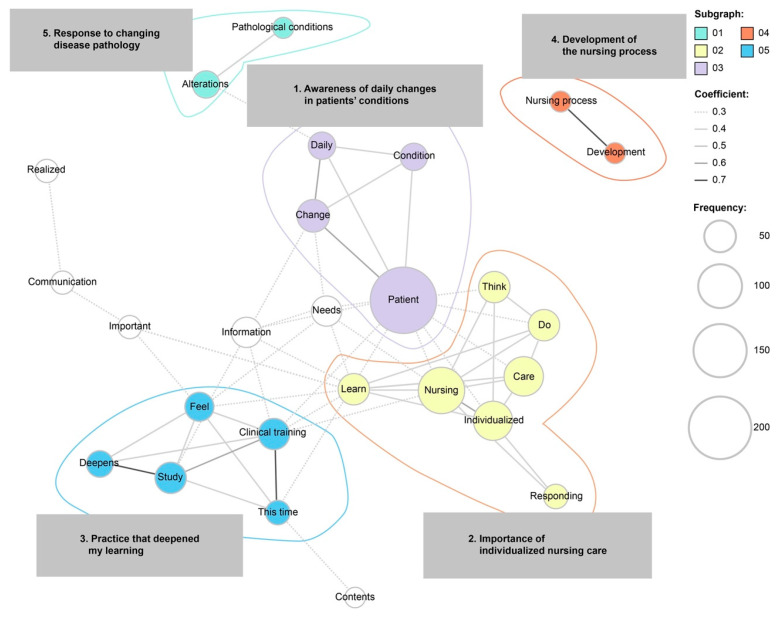
Co-occurrence network diagram of what students learned more about from simulation-based clinical training.

The responses included 232 sentences and 3265 words in 643 categories. Jaccard coefficients were calculated using these data, and the co-occurrence network is shown in Figure 1 (subgraph detection by mediation).

There were five subgraphs in the co-occurrence network diagram: (1) awareness of daily changes in patients’ conditions, (2) the importance of individualized nursing care, (3) practice that deepened my learning, (4) development of the nursing process, and (5) response to changing disease pathology.

### 3.2. Students’ Perspectives on Clinical Training Benefits

A total of 3044 words were extracted from responses to the benefits of the training, of which 150 frequently mentioned words were selected. The top five words were patient (118), group (62), actual (60), practice (27), and care (48) (Table 3).

**Figure 2:**
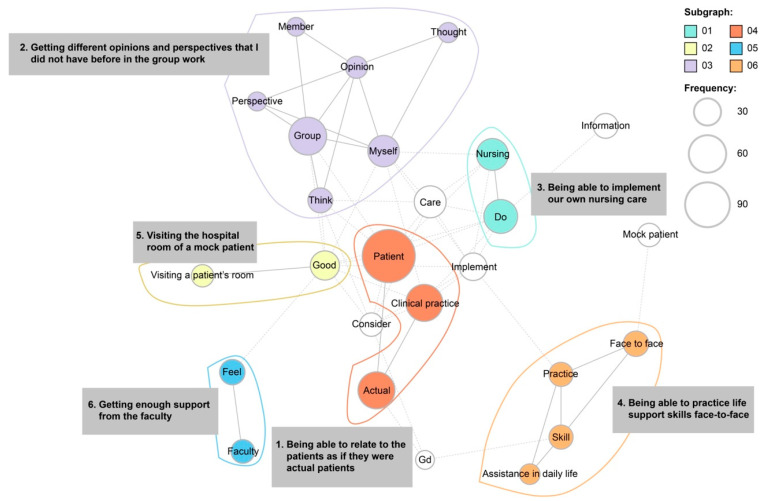
Co-occurrence network diagram of students’ perspectives on benefits of the clinical training.

In total, 208 sentences were received as responses for the benefits of the simulation-based training, from which 3029 words belonging to 678 categories were extracted. The co-occurrence network diagram comprised six subgraphs (Figure 2). Hence, students’ responses could be categorized as (1) being able to relate to the patients as if they were actual patients, (2) getting different opinions and perspectives in the group work that I did not have before, (3) being able to implement our own nursing care, (4) being able to practice life support skills face-to-face, (5) visiting the hospital room of a mock patient, and (6) getting enough support from faculty.

### 3.3. Difficulties Encountered with the New Practice Format

The total number of words extracted for “difficulties encountered” was 2665. Of the 150 most frequently occurring words selected, “patient” (72), “feeling” (57), “time” (49), “information” (42), and “difficult” (42) were the top 5 (Table 4).

**Figure 3:**
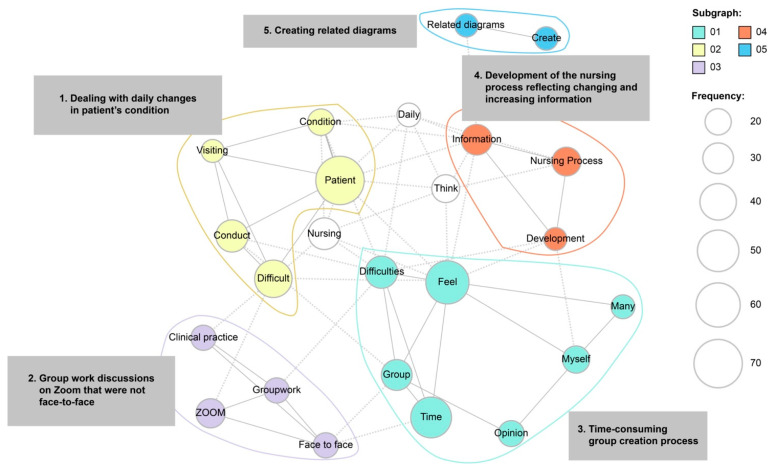
Co-occurrence network diagram of difficulties encountered with the new practice format.

Students explained the challenges they encountered using 214 sentences. We extracted 2665 words from 740 categories to create the co-occurrence network, as shown in Figure 3. The difficulties experienced by students could be categorized into five groups: (1) dealing with daily changes in a patient’s condition, (2) group work discussions on Zoom, (3) time-consuming group creation process, (4) development of the nursing process reflecting change and increasing information, and (5) creating related diagrams.

## 4. Discussion

### 4.1. Students’ Comprehension and Motivation for Clinical Practice

A high percentage (80.5%) of students indicated that they felt this training would be useful in a genuine clinical situation. Furthermore, 94.3% of the students were motivated to continue their studies and pursue nursing after the two-week simulated training. These findings suggest that simulation-based learning using mock patients may positively impact self-efficacy and motivation to learn, thus enhancing knowledge and clinical skills.

Our findings are consistent with those of previous studies [6,10] showing that simulation-based learning with mock patients may benefit knowledge acquisition, communication skills, self-efficacy, learning motivation, and clinical skill acquisition. Further, our results support the theory that the simulated patient pedagogy can be applied in educational settings as an active learning methodology if appropriately incorporated by considering the readiness of the target population.

Words such as “change” and “individuality” were most frequently mentioned, indicating what participants learned more deeply and in which areas they gained a deeper understanding.

Sub-categories related to “change” were “awareness of the daily changes in the patient’s condition” and “response to changing pathologies”. Prior to this training, nursing students’ practice had been conducted using a cross-sectional case study (traditional, static case study); thus, the students’ understanding of the changes in the patient’s condition was limited. Through the unfolding case study used in this training, students noticed the daily changes in the patient’s condition. They were also aware of the changes in the patient’s feelings and the needs associated with their condition. Prior research has illustrated that unfolding case studies can develop students’ critical thinking skills in areas such as information seeking, logical reasoning, and clinical data analysis more effectively than traditional static case studies [11]. Additionally, most participants (97.6%) stated that they could understand the changes in the patient’s condition over time through the simulation. This result suggests that the difficulty level of the case study and the amount of information provided with the simulated electronic medical records were sufficient to gain an appropriate understanding of the simulated case. Further, the simulated case (community-acquired pneumonia) used in this training and the extent of the information provided to students were considered appropriate for their learning potential as second-year students.

Individualized nursing care was another important concept that students learned during simulation-based learning. Nursing care of this nature indicates quality care, as it enhances positive patient outcomes [12,13]. Thus, the importance of individualized care in nursing education and patient care is now being emphasized within policies [14,15]. Personalized care involves the planning and practice of nursing care according to individual patient characteristics, demands, preferences, experiences, feelings, perceptions, and opinions [12,13,16]. In this study, the students could grasp the importance of individualized care by experiencing and understanding the mock patient’s social background, living conditions, and preferences before hospitalization.

Additionally, students stated that they experienced a profound understanding of the “development of the nursing process”, indicating that they recognized the importance of individualized nursing care and the necessity of fully understanding and effectively implementing the nursing process to practice such care. This may be because all students could discuss and exchange opinions through group work, and instructors could take their time and guide and instruct the students, all of whom were following the same case study. In hospital-based clinical practice, students are assigned to patients with different medical conditions and socio-demographic backgrounds; therefore, not all cases are appropriate for every student’s knowledge and skill level. Using a single case is less likely to lead to differences between the teacher and the learner.

Nurses should be experienced in effectively conducting the nursing process and developing individualized nursing care practices to provide quality nursing care [17]. Nursing students typically rely on textbook nursing care plans, and therefore creating a challenge for first-time students to develop an individualized patient nursing process is necessary. Nursing students tend to link theoretical knowledge to clinical practice in a non-critical-thinking manner. Therefore, through this program, they recognize the importance of applying previously learned theoretical knowledge to individual nursing care practice [18,19,20] to enhance professional development. Benner’s Novice to Expert Model states that novice nursing students must be taught about individualized nursing, integrate this knowledge into their practice, and be given the responsibility to plan and implement nursing care for patients whose conditions are expected to change [18]. Although this training was simulation-based, students may have acquired a greater understanding of a few aspects of the clinical setting culture, the outcomes expected of them as nursing students, and gained experience implementing individualized patient care.

### 4.2. Students’ Perspectives on “Benefits of the Simulation-Based Training”

The most frequently used words regarding the “benefits of this training” included “patient”, “group”, “actual”, “practice”, “care”, and “learning from the group work”. The sub-categories identified were “being able to interact with patients as if they were actual patients”, “being able to implement our own nursing care”, “being able to practice life support skills face-to-face”, and “being able to visit the simulated patients’ hospital rooms”. A simulation that closely resembled a clinical situation was implemented, referring to the four phases of the clinical decision-making model: “noticing”, “interpreting”, “responding”, and “reflecting”. Students were exposed to various situations, reflected on those situations through debriefing, conceptualized their own experiences, identified gaps between expected outcomes and performance, and clarified issues. The training program was designed to assist students in performing better in unfolding situations. It was expected that through this simulation-based practice, students would experience how to build a relationship with a patient, practice life-support care, and create a nursing care plan on their own. Cant and Cooper [21] stated that Kolb’s [22] experiential learning theory, which is the basis for simulation learning, can narrow the gap between “knowing” and “being able to do”. Simulation-based learning is beneficial in nursing education [23,24,25,26], and recent studies have shown benefits such as improved nursing competence, self-efficacy, and learning satisfaction [27,28,29].

Moreover, “adequate support from faculty” was a positive aspect of this training. The high-fidelity simulations enabled teachers to replicate several patient scenarios, enabling students to learn and practice nursing skills (cognitive, motor, and critical thinking) in an environment where patients were not at risk. Furthermore, during debriefing sessions, students reflected on each scenario and clarified issues objectively.

A U.S. study found no significant differences in clinical competence, nursing knowledge, and NCLEX^®^ pass rates between students who learned through traditional clinical practice and those for whom simulations replaced some clinical time [30]. Nurse managers’ ratings of clinical competence and readiness to practice also showed no significant differences after six weeks, three months, or six months of employment. High-fidelity simulation reduces student anxiety and increases confidence in nursing tasks and patient management. Therefore, the high-fidelity simulation-based learning implemented in this study may increase students’ confidence in future clinical nursing practices. To respond to unforeseen circumstances, it is important to establish a new form of clinical practice that is integrated with high-fidelity simulation, thereby decreasing dependence on medical institutions. Furthermore, group work exposure (including online) allowed different opinions and perspectives to be experienced.

This study led to the discovery of new perspectives and a greater openness toward others’ opinions. A previous study that conducted intra-professional team training using high-fidelity simulation has shown the benefits of teamwork regarding role recognition and clarification, adaptation to the team environment, and a sense of professional solidarity [30]. Even with remote training, it is possible to construct programs with high learning effectiveness while incorporating active learning techniques.

### 4.3. Difficulties Encountered by Students during Training

The students used the terms “patient”, “feeling”, “time”, “information”, and “difficult” frequently when describing the challenges faced during the training, indicating the difficulty in dealing with patients and organizing information over time.

The sub-categories identified through the co-occurrence network diagram were “dealing with daily changes in patient’s condition”, “group work discussions conducted in a non-face-to-face format”, “time-consuming group work”, “developing the nursing process reflecting increasing information”, and “creating related charts”. Several comments indicated a sense of difficulty in developing the nursing process owing to changes in the patient’s condition.

However, several challenges discussed above, such as “awareness of daily changes in the patient’s condition”, “responding to changing conditions accordingly”, and “development of the nursing process”, were also discussed as issues the students experienced during this training. This suggests that the students deepened their learning even while experiencing difficulties.

Unlike conventional case studies, which provide students with all the relevant information, unfolding case studies intentionally create less-than-perfect situations. This facilitates the development of critical thinking skills in students, such as information seeking, logical reasoning, and analysis of clinical data [31,32].

A previous study has shown that developmental case studies more accurately convey the reality of clinical practice because nurses tend to present clinical information in phases rather than simultaneously [33]. The study suggested that unfolding case studies increase students’ engagement, self-esteem, and preparation for the role of a professional nurse. Furthermore, unfolding case studies enable students to reflect on their strengths and limitations identified during the learning process, which can adequately prepare them for future clinical experiences [34]. Unfolding case studies that evolve with time may facilitate the connection between theory and practice, as novice nursing students can experience challenging situations in realistic environments while increasing relevant knowledge and skills.

The most common training obstacles were “group work discussions conducted in an online format” and “the time-consuming group creation process”. Mukhtar et al. [35] stated that the advantages of online learning include the ability for students to study at any time, independently, and increase their study time by eliminating the commute to and from school. However, several limitations of online learning have been reported, including limited student interaction, inability to provide adequate feedback from faculty, and reduced student engagement [36,37]. In the current clinical training, students may have experienced difficulties with two-way communication, reduced motivation, and decreased attention span when participating in online group work at home. However, online learning was an essential educational tool during the pandemic and has worked well in many areas. More detailed attention to time allocation and assignment load may be needed while considering ways to effectively merge face-to-face and remote group work.

Regarding practical applications, in traditional clinical practicum, achieving standardization of case complexity across a wide range of patient cases is challenging. Educational effectiveness may vary depending on factors such as clinical facility policies and the instructor’s educational background, potentially leading to inconsistencies in learning outcomes. In contrast, simulation-based practicum, as conducted in this study, replicates real clinical scenarios with simulated patients in a controlled environment. This allows students to practice clinical skills, decision-making, and critical thinking safely before interacting with real patients. Additionally, nursing students can learn from failures without compromising patient safety, thus providing a safe environment for skill refinement. Standardization and reproducibility of clinical scenarios will ensure that all students receive consistent learning experiences. Simulation scenarios can be carefully designed and implemented to address specific learning objectives and competencies. In an era where predicting the future amidst diverse and complex events such as large-scale disasters or conflicts is increasingly challenging, combining simulation-based simulated patient practicum may help maintain the quality of education consistently [38].

### 4.4. Limitations

This study was conducted on students at a single university in Japan and based on a limited sample size, making the generalization of the results difficult. Furthermore, as this was a cross-sectional examination of one practical training course, it is necessary to conduct a longitudinal study to determine the extent to which clinical skills and competencies are affected at the time of graduation or after graduation. Further studies can consider linking examination results to student perceptions to avoid possible response bias, as well as employing a larger, more diverse sample of students for generalizability.

## 5. Conclusions

In traditional clinical practicum, standardizing case complexity across various patient cases is difficult and may lead to inconsistent educational effectiveness due to factors like facility policies and instructor backgrounds. Simulation-based practicum, as seen in this study, offers a controlled environment to replicate real clinical scenarios with simulated patients, allowing students to safely practice their clinical skills and decision-making before engaging in real patient interactions. This approach enables learning from failures without risking patient safety, ensuring standardized learning experiences for all students. Carefully designed simulation scenarios can address specific learning objectives and competencies, potentially contributing to maintaining consistent educational quality, especially in unpredictable times such as during large-scale disasters or conflicts.

## Figures and Tables

**Table 1:** Distribution of responses to multiple-choice items.

		*n*	%
Were you able to understand the changing conditions of patients over time?			
	1. I understood very well	60	49.2
	2. I could understand it well	60	49.2
	3. Neither	1	0.8
	4. Did not understand much	1	0.8
	5. Did not understand at all	0	0.0
Do you think the training would be useful in actual clinical situations?			
	1. Very useful	99	81.1
	2. A little useful	23	18.9
	3. Neither	0	0
	4. Not so useful	0	0
	5. Not at all useful	0	0
Did it motivate you to continue studies and pursue nursing in the future?			
	1. Very well done	92	75.4
	2. Well done	23	18.9
	3. Done	6	4.9
	4. Needs effort	1	0.8
	5. Not possible	0	0

**Table 2:** Top 25 most frequently mentioned words for “what they learned more about or got a deeper understanding of through the training”.

Extracted Terms	Number of Occurrences	Extracted Terms	Number of Occurrences
Patient	209	Daily	35
Nursing	97	Learning	33
Care	74	Assistance	29
Individuality	73	Deepens	28
Change	53	Valued	27
Learn	51	Important	25
Need	48	Communication	24
Think	46	Respond	24
Do	46	Understand	24
Information	42	Alterations	22
Practice	41	This time	21
Feel	37	Think	21
Condition	37		

**Table 3:** Top 25 most frequently used words for “positives of the training”.

Extracted Terms	Number of Occurrences	Extracted Terms	Number of Occurrences
Patient	118	Skill	24
Group	62	Feel	23
Actual	60	Information	23
Clinical practice	58	Faculty	23
Care	48	Practice	22
Do	47	Be completed	22
Myself	43	Mock patient	21
Nursing	40	Opinion	19
Good	34	Be able to perform	19
Implement	32	Assistance	17
Think	25	Visiting a patient’s room	18
Thought	25	Get	17
Face to face	25		

**Table 4:** Top 25 most frequently used words for “difficulties encountered during the training period.”

Extracted Terms	Number of Occurrences	Extracted Terms	Number of Occurrences
Feel	57	Opinion	18
Time	49	Face to face	18
Information	42	Clinical practice	17
Difficult	42	Many	17
Conduct	31	Development	17
Nursing	30	Related diagrams	16
Difficult	30	Myself	16
Groups	29	Create	15
ZOOM	24	Daily	15
Nursing Process	22	Visiting a patient’s room	15
Thinking	20	Think	14
Condition	19	Need	14
Group Work	18		

## Data Availability

The data are not publicly available due to ethical restrictions.

## Supplementary Materials

The following supporting information can be downloaded at https://www.mdpi.com/article/10.3390/nursrep14020074/s1.

## Public Involvement Statement

There was no public involvement in any aspect of this research.

## Guidelines and Standards Statement

This manuscript adheres to STROBE guidelines for observational studies.
