# Supplementary material for: Perspectives of Nursing Students on Hybrid Simulation-Based Learning Clinical Experience: A Text-Mining Analysis
Source: Nurs Rep. 2024 Apr 18;14(2):988–99. doi: 10.3390/nursrep14020074 (PMC11036196; doi:10.3390/nursrep14020074)
Supplement: Supplementary file 1 [file nursrep-14-00074-s001.zip › nursrep-2929380-supplementary.pdf]

nursing reports

MDPI

Supplement 1. Questionnaire

(1) Could you understand the changes in the patient's condition over time through the simulated patient case?
    1. I understood very well
    2. I could understand it well
    3. Neither
    4. Did not understand much
    5. Did not understand at all

(2) Do you think this training will be useful in clinical practice?
    1. Very useful
    2. A little useful
    3. Neither
    4. Not so useful
    5. Not at all useful

(3) Did it motivate you to learn nursing and to study more in the future?
    1. Very well done
    2. Well done
    3. Done
    4. Needs effort
    5. Not possible

(4) What did you learn more about or got a deeper understanding of through the training
    【Free notes in 400 words or less】

(5) What was the positives of the training
    【Free notes in 400 words or less】

(6) What was the difficulties encountered during the training period
    【Free notes in 400 words or less】

(7) Please feel free to describe any impressions you may have through the practical training.
    (This is not related to the evaluation, so please feel free to describe it as you like.)
    【Free notes in 500 words or less】

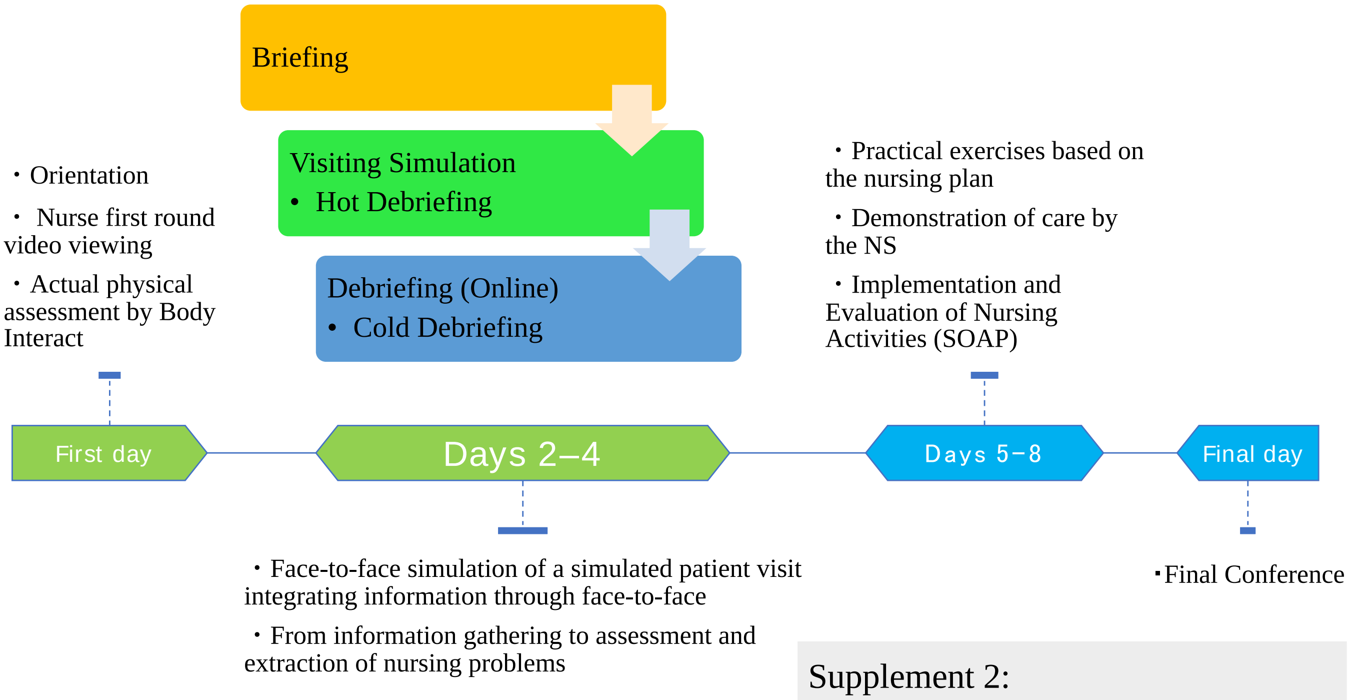

Supplement 2:
Outline of the Practicum Schedule
